# Scientometric analysis of trends in research in laryngopharyngeal reflux

**DOI:** 10.1007/s00405-025-09381-1

**Published:** 2025-04-07

**Authors:** Dhanshree R. Gunjawate, Rohit Ravi

**Affiliations:** Department of Audiology and Speech-Language Pathology, Kasturba Medical College Mangalore, Manipal Academy of Higher Education, Manipal, India

**Keywords:** Laryngopharyngeal reflux, Scientometric analysis, Voice disorders, Reflux

## Abstract

**Objective:**

Scientometric analysis helps to understand the research trends and identify top contributors, publishers, trends, and research topics. Laryngopharyngeal reflux has been a topic of interest in research and clinical practice. The literature on laryngopharyngeal reflux is evolving and a scientometric analysis will help us understand the trends better. The present study aimed to analyze published data to identify trends, top contributors, top institutions and countries, growth of publications, keywords and keyword co-occurrence.

**Methods:**

The present study aims to analyze the evolution of Laryngopharyngeal reflux research using a scientometric analysis approach for publications from the Scopus database using keywords related to laryngopharyngeal reflux. Documents related to laryngopharyngeal reflux were identified for further analysis. Microsoft Excel and VosViewer were used to perform bibliometric analysis.

**Results:**

A total of 7,327 hits were obtained, and after applying filter, 5637 studies were retained for further analysis. The results revealed an increase in publications on LPR, which surged after the early 2000s. The Unites States had the maximum research output, and the Journal of Voice had the highest number of publications. The keyword co-occurrence analysis helped identify five research themes on laryngopharyngeal reflux.

**Conclusion:**

The findings reveal a rising trend in laryngopharyngeal reflux, especially in recent years. The presence of international collaborations and a high volume of research will help bridge gaps, enable capacity building and improve understanding of LPR.

**Supplementary Information:**

The online version contains supplementary material available at 10.1007/s00405-025-09381-1.

## Introduction

Laryngopharyngeal reflux (LPR) is a complex disease characterized by a variety of symptoms and clinical presentations often influenced by direct and indirect factors [1]. The clinical presentation of LPR includes throat clearing, chronic cough, something stuck in the throat/globus sensation, excess throat mucus, bad breath, hoarseness, pain in the throat, post-nasal drip and dysphonia [2–5].

LPR was initially considered an extension of Gastroesophageal Reflux disease (GERD); however, the differences in the mechanisms, patterns, manifestations, and treatment led to the evolution of a separate condition [6]. Patients with LPR exhibit more head and neck symptoms, predominantly while upright, and have normal esophageal motility. Esophagitis is one of the most important diagnostic indicators for GERD and is uncommon in individuals with LPR. LPR is commonly associated with the etiology of several laryngeal conditions, namely contact ulcers, granulomas, laryngeal carcinoma, reflux laryngitis, subglottic stenosis, and vocal nodules [6–8].

A systematic review has revealed the increasing prevalence of GERD and LPR by 4% each year since 1976 [9]. It is estimated that LPR is prevalent in over 50% of patients who complain of dysphonia [10] or attend an otolaryngology clinic [11]. The diagnosis of LPR has always been a challenge for the healthcare fraternity as compared to GERD. LPR is clinically detected using laryngoscopy to detect mucosal injury, laryngeal irritation and inflammation [7, 12], and pH monitoring [13]. No gold standard exists for detecting LPR, which continues to pose a significant challenge to its accurate diagnosis and management [14].

The management of LPR involves use of medical treatment along with lifestyle modifications, such as behavioural and dietary changes [15, 16]. Dietary modifications include excluding acidic food items, alcohol, caffeine, chocolates, fats, late-night meals and spicy food items [16, 17]. However, robust studies to support effect of diet modification alone is lacking [16]. Medical treatment includes using proton pump inhibitors, prokinetic agents, H2-receptor antagonists and mucosal cytoprotectants [2, 18].

LPR remains a challenge to clinicians and patients due to its generalized non-specific symptoms, lack of standardized testing, poor understanding of the pathophysiology, difficulty with diagnosis, multiple associated factors, and lack of standardized treatment [18]. Thus, the interest in the diagnosis and management of LPR continues, and each year, we witness a growth in publications that improve the understanding of this condition. While work has been published using secondary data analysis methods such as scoping reviews, systematic reviews and analysis of content available on YouTube, there have been no scientometric explorations related to LPR. A recent publication was the first bibliometric analysis of literature on refractory GERD. Their findings contributed significantly to the understanding of refractory GERD by providing research insights, focus, and hot topics [19]. A review of publications in pediatric LPR in the last decade highlighted the increase in publications and the need for more advanced research to better understand pediatric LPR [20]. However, to date, no bibliometric analysis has been conducted focusing on LPR.

Therefore, the aim of the present study is to analyze the published data using scientometric analysis to examine the evolution of research on LPR, key researchers, institutions and countries, research priorities, and co-occurrence of keywords.

## Method

Scopus, managed by Elsevier, is the largest comprehensive electronic academic database, with advanced functionalities such as unique identifiers for authors and affiliations. It provides comprehensive author metrics, like h-index, citation counts, and subject areas, while also enabling article retrieval by author, source, or citation and set up alerts. Journals are accessible using SCImago Journal Rank, Source Normalized Impact per Paper, and citation analysis [21, 22].

### Data sources, keywords and search strategy

Scientific research in LPR was retrieved from the Scopus electronic database. Keywords such as ‘laryngopharyngeal reflux disease’, ‘reflux laryngitis’, ‘laryngeal reflux’, ‘gastropharyngeal reflux’, ’pharyngoesophageal reflux, ‘supraesophageal reflux’, ‘extraesophageal reflux’, ‘atypical reflux’, ‘laryngopharyngeal reflux were combined using ‘OR’ Boolean operator and searched on Scopus on 17th January 2025.

Details about the search strategy have been described in Appendix − 1.

### Study selection criteria

The initial search was carried out without any filters to identify a broad range of studies. After the initial search, filters were added for year, language, document, and source type. For the final analysis, studies published up to 2024 in the English, including reviews, articles, and conference papers from two source types (journals and conference proceedings), were included.

### Data and bibliometric analysis

The data was exported from Scopus to Microsoft Excel and VOS viewer. Scopus analysis and Microsoft Excel were used to identify the top 10 authors, organizations/institutions, countries, and journals. VOS Viewer was used for visualisation and bibliometric analysis. Keyword analysis was carried out to identify the most researched keywords and themes. Common keywords (provided in the results) were excluded during visualisation and bibliometric analysis.

## Results

A total of 7,327 hits were obtained for the initial search before applying any filters. The top three publication languages were English (6581, 89.82%), Chinese (199,2.72%), and German (170,2.32%). In terms of the document type, 4869 (66.45%) were articles, 1340 (18.29%) reviews, 414 (5.65%) book chapters, 220 (3%) letters to editor, 159 (2.17%) conference proceedings, 112 (1.53%) note, 110 (1.5%) editorials, 59 (0.81%) short surveys, 36 (0.49%) books, 5 (0.07%) retracted papers and 3 (0.04%) erratum.

After applying filters of publication year, language, document and source type, 5637 studies were retained for further analysis. Of these, 5615 (99.61%) were journal articles and 22 (0.37%) conference proceedings. With respect to document type, 4321 (76.65%) were articles, 1190 (21.11%) were reviews, and 135 (2.39%) were conference proceedings.

### Analysis of countries and institutions

**Fig. 1:**
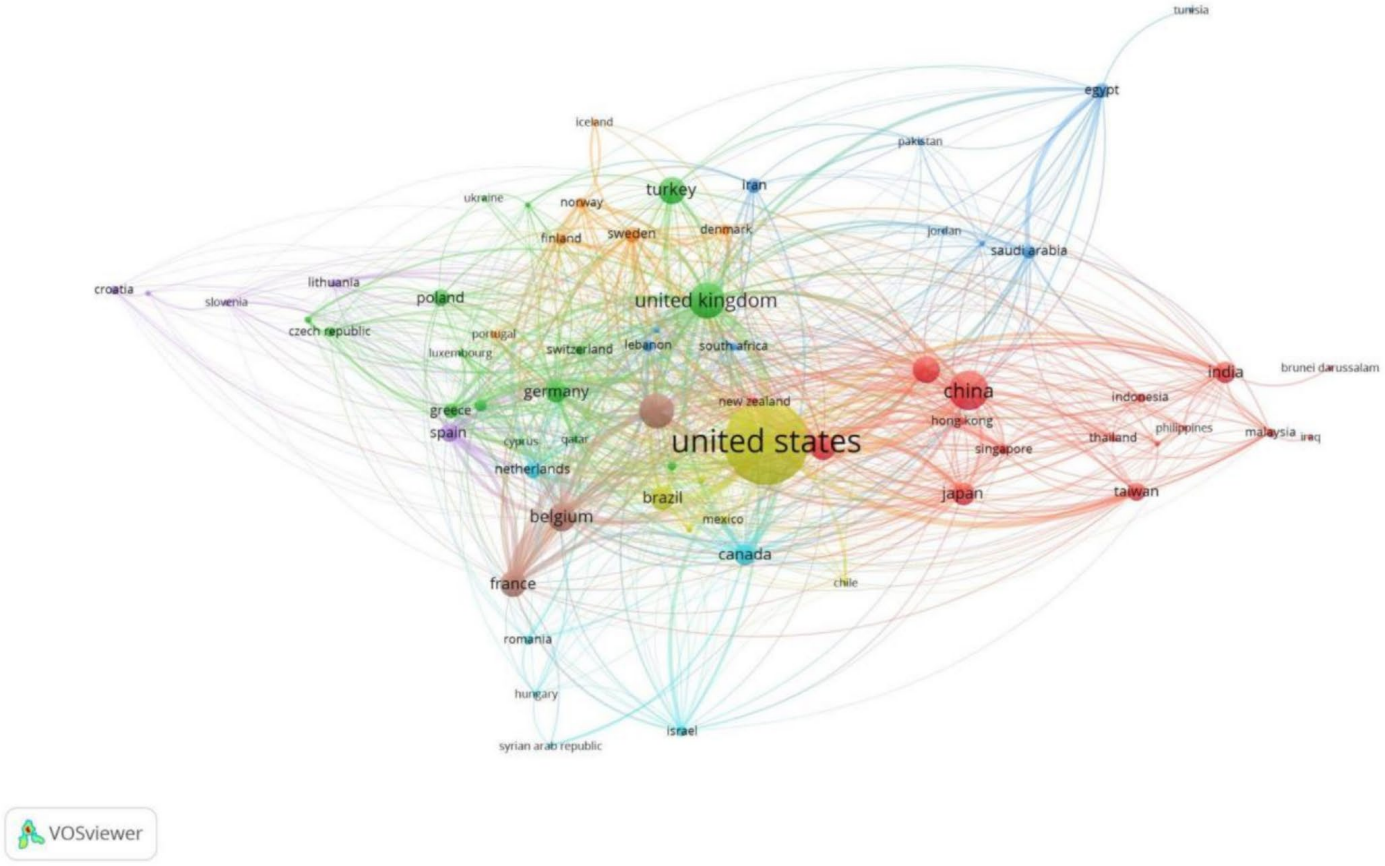
Visual representation of collaboration between countries

As presented in Table 1, a total of 107 countries have been involved in research in LPR, with the United States of America (USA) (39.56%) contributing the maximum number of papers and collaborations. Figure 1 depicts the visual representation of collaborations between the countries. The size of the circle depicts the weight of the item, in this case, the number of collaborations and the thickness of the lines indicate the number of collaborations, with thicker lines indicative of more collaborations.

**Table 1:** List of top 10 countries along with number and percentage of records

Country	*n* (%) of records
USA	2230 (39.56%)
China	494 (8.76%)
United Kingdom	421 (7.47%)
Italy	387 (6.87%)
Belgium	252 (4.47%)
South Korea	234 (4.15%)
Turkey	225 (3.99%)
Australia	215 (3.81%)
France	207 (3.67%)
Brazil	187 (3.32%)

Table 2 depicts the top 10 universities/institutions contributing the most publications, along with the number and percentage of records. The Medical College of Wisconsin, Milwaukee, Wisconsin, USA, emerged as the top contributor with 147 records, followed by Université de Mons, Mons, Belgium, with 130 records.

**Table 2:** List of top 10 universities/institutions contributing most publications along with number and percentage of records

University /Institution	*n* (%) of records
Medical College of Wisconsin (Milwaukee, Wisconsin, USA)	147 (2.61%)
Université de Mons (Mons, Belgium)	130 (2.31%)
Harvard Medical School (Boston, Massachusetts, USA)	108 (1.92%)
Vanderbilt University Medical Center (Nashville, Tennessee, USA)	106 (1.88%)
Hopital Foch (Suresnes, France)	100 (1.77%)
Centre Hospitalier Universitaire Saint Pierre (Brussels, Belgium)	99 (1.76%)
Université Paris-Saclay (Paris, France)	93 (1.65%)
Northwestern University Feinberg School of Medicine (Chicago, Illinois, USA)	85 (1.51%)
University of Wisconsin School of Medicine and Public Health (Madison, Wisconsin, USA)	79 (1.40%)
Drexel University College of Medicine (Philadelphia, Pennsylvania, USA)	72 (1.28%)

### Analysis of journals

The top 10 journals publishing research on LPR have been tabulated in Table 3, along with details such as publisher and number of records. A strong preference towards publishing in journals that are focused on laryngology and voice and thereby having a higher viewership among laryngologists and speech-language pathologists (SLPs), can be noted. This is reflective of the relevance of LPR research to these two fields and the specialized nature.

**Table 3:** List of top 10 journals for publishing papers on LPR

Name of Journal	Publisher	*n* (%) of records
Journal of Voice	Elsevier	362 (6.42%)
Laryngoscope	Wiley	297 (5.27%)
Otolaryngology-Head and Neck Surgery	Wiley	185 (3.28%)
European Archives of Oto Rhino Laryngology	Springer	169 (3.00%)
Annals Of Otology, Rhinology and Laryngology	SAGE	133 (2.36%)
International Journal of Pediatric Otorhinolaryngology	Elsevier	85 (1.51%)
Ear Nose and Throat Journal	SAGE	82 (1.45%)
Journal of Laryngology and Otology	Cambridge University Press	81 (1.44%)
Current Opinion in Otolaryngology and Head and Neck Surgery	Lippincott Williams & Wilkins	74 (1.31%)
American Journal of Gastroenterology	Lippincott Williams & Wilkins	69 (1.22%)

### Analysis of authors

A total of 159 authors were identified as contributors to the research in LPR. Table 4 depicts the top 10 contributing authors and their contributions. Dr Jérome R. Lechien emerged as the top contributor with 142 published works.

**Table 4:** Top 10 contributing authors

Name of author	Country	*n*(%) of records
Lechien, J.R.	Belgium	142 (2.52%)
Vaezi, M.F.	USA	102 (1.81%)
Sataloff, R.T.	USA	77 (1.37%)
Saussez, S.	Belgium	73 (1.30%)
Johnston, N.	USA	62 (1.10%)
Fass, R.	USA	45 (0.80%)
Carroll, T.L.	USA	41 (0.73%)
Hans, S.	France	41 (0.73%)
Belafsky, P.C.	USA	39 (0.69%)
Koufman, J.A.	USA	39 (0.69%)

### Growth of publications

**Fig. 2:**
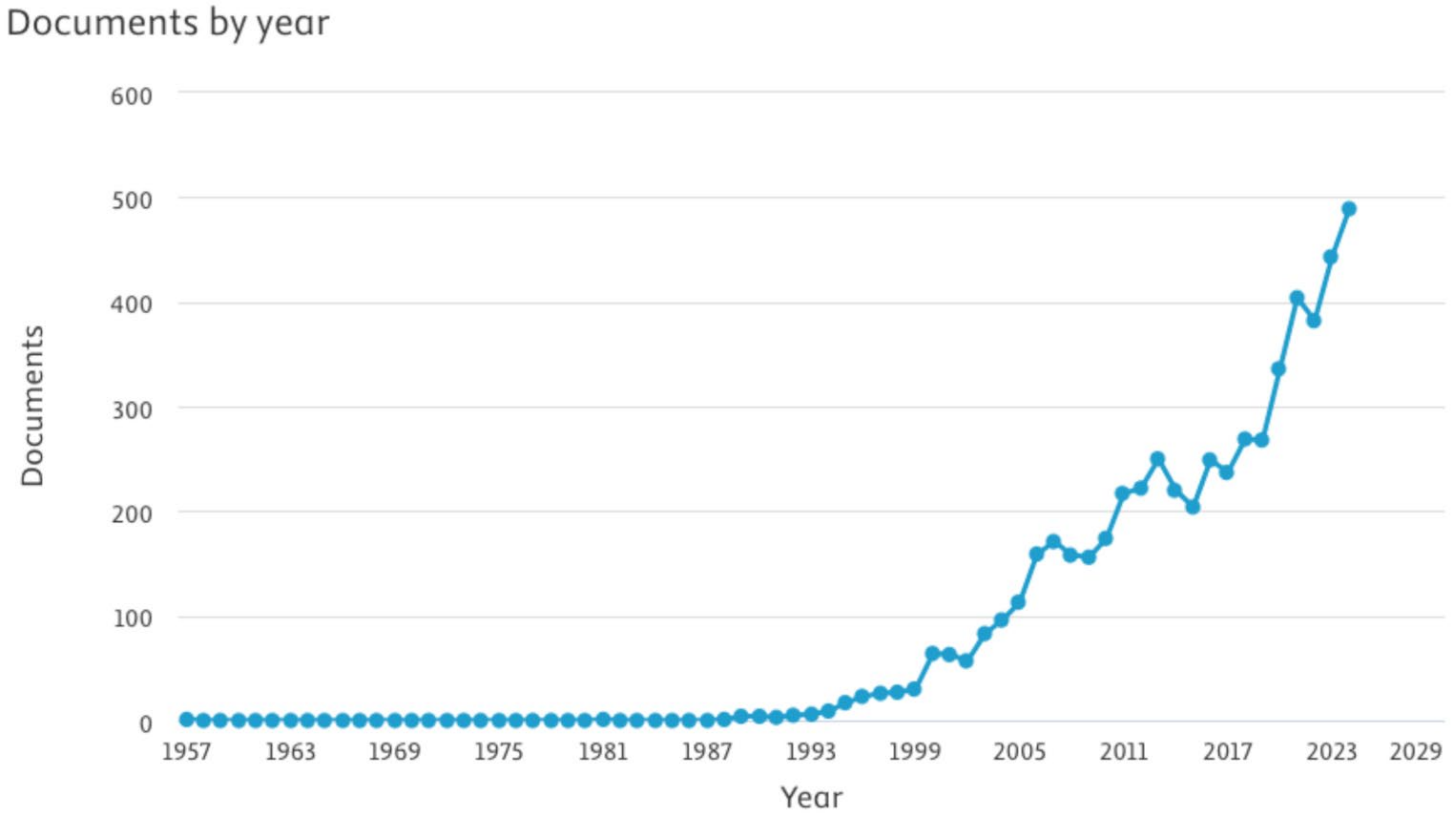
Growth in the number of publications from 1957 to 2024

The distribution of growth of publications is presented in Fig. 2, which clearly depicts the continuous increase in research publications each year, especially after the early 2000s.

**Fig. 3:**
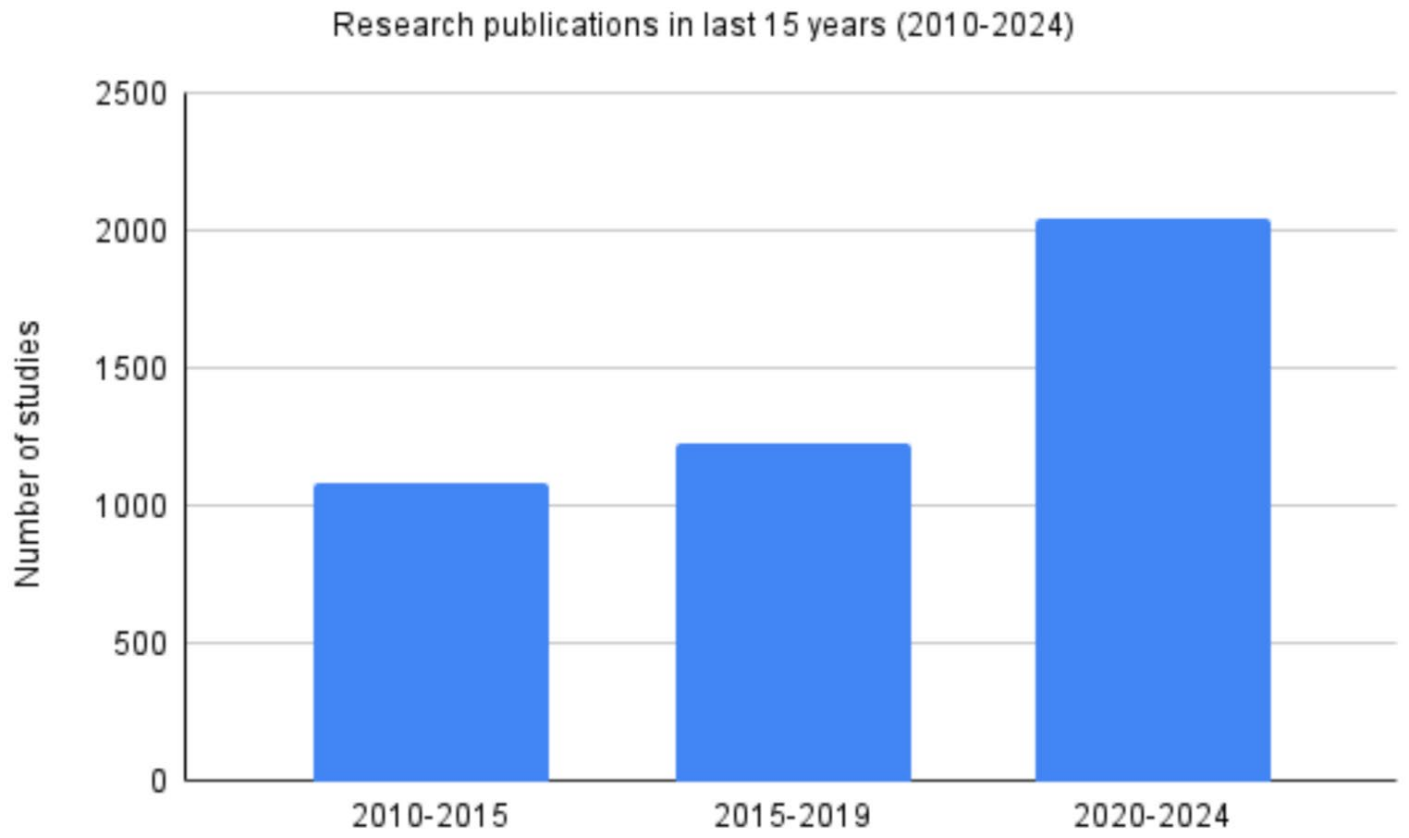
Research publications in the last 15 years (2010–2024)

A moderate rise in publications can be noted between 2000 and 2010, with publications increasing from 64 to 174 which was followed by a period of acceleration from 174 in 2010 to 336 in 2020. The period between 2020 and 2024 saw the most rapid rise in LPR publications, highlighting the heightened research and clinical interests. A closer look at the research output for the last 15 years also reveals increased interest in LPR, as seen in Fig. 3.

### Keyword analysis and keyword co-existence analysis

The analysis of keywords and their co-existence is a crucial aspect of scientometric analysis and helps to identify key topics and potential areas for continuing research. A keyword co-existence analysis was conducted to identify the most common research areas and themes. Common keywords such as children, impedance, prevalence, pediatric, systematic review, meta-analysis, questionnaire, and child were excluded.

**Fig. 4:**
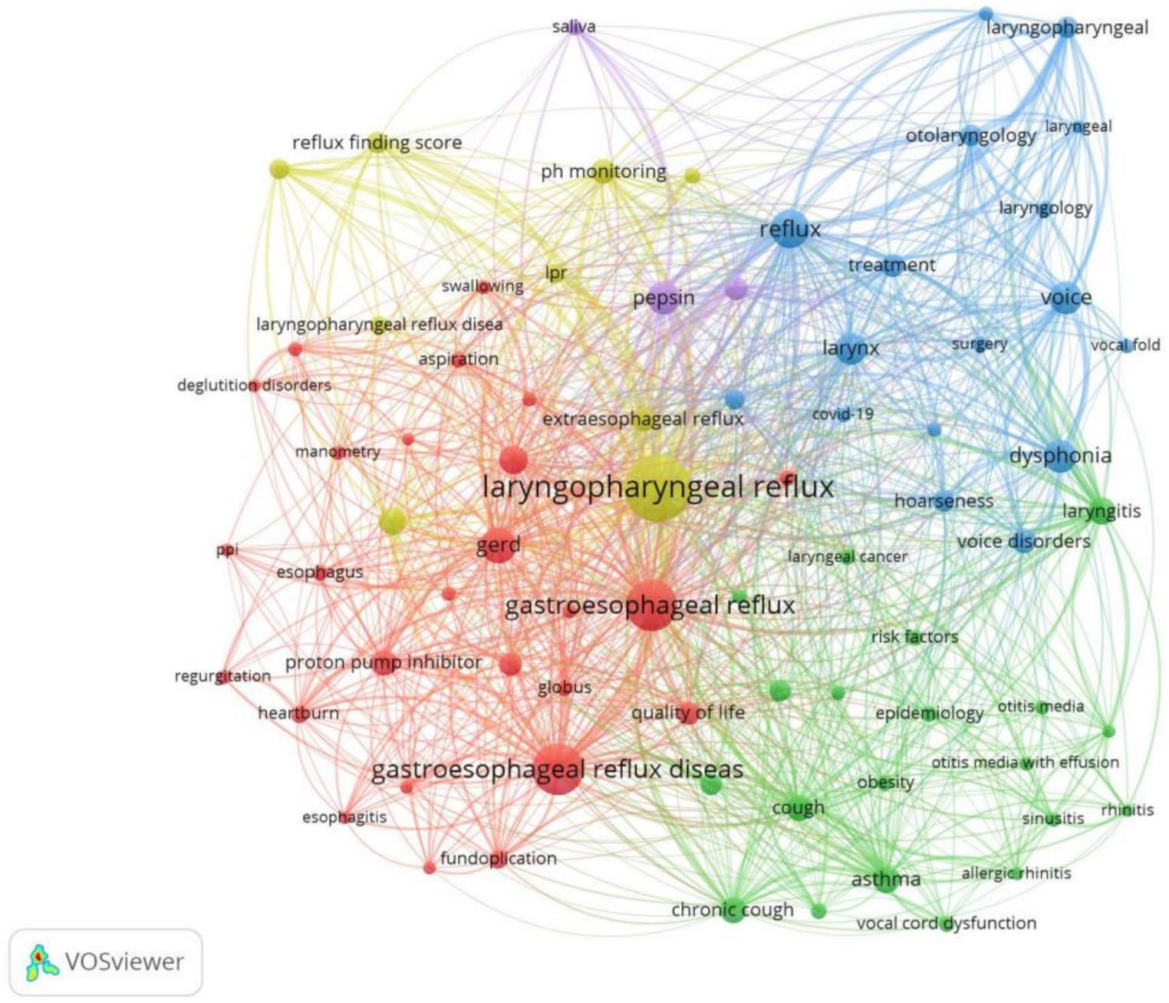
Co-occurrence for top 75 author keywords

Using clustering, the co-occurrence of keywords could be divided into five clusters depicted by red, green, blue, yellow and purple. The red cluster contained keywords related to ‘GERD, LPR and medical treatment’, while the green cluster contained keywords on ‘associated health conditions and symptoms’. The blue cluster included ‘voice and larynx-related’ keywords, while the yellow cluster included ‘diagnostic and assessment techniques’. The purple cluster was the smallest and included keywords related to the ‘digestive system’.

## Discussion

The present study provides a scientometric analysis of the literature on LPR up to 2024, insights related to the top 10 countries/institutions, journals and authors, publication trends and keyword analysis.

Regarding LPR research, the top contributor and collaborator is the USA (39.56%). The USA was also reported to have the highest number of publications in a scientometric study on Refractory GERD [19]. The top contributing institute for LPR research was the Medical College of Wisconsin, Milwaukee, Wisconsin, USA. The dominance that the US enjoys throws light on the crucial role being played in shaping the research landscape for both LPR and GERD research. The collaborations established, and the high volume of publications have contributed significantly to the overall understanding of the condition. Such international collaborations, especially with low- and middle-income countries, will help overcome gaps, help capacity building, and improve the overall understanding of the condition [23, 24]. It is crucial to note the growing contribution from other countries such as China, UK and European countries such as Italy and Belgium. This shift is crucial from research and clinical perspectives by providing global overview on the diagnosis and management.

In terms of the authors, Jerome R. Lechien, an otolaryngologist researcher from Belgium, has the highest number of publications on LPR. He has been instrumental in conducting several international collaborative initiatives to explore the global practices related to LPR as well as establishing consensus frameworks [25–27]. Michael F Vaezi, a gastroenterologist researcher from the USA, had the second-highest publications, closely followed by Robert T. Sataloff, an otolaryngologist researcher from the USA.

Journal of Voice is the most common choice for publishing articles on LPR. The Journal of Voice, published by Elsevier, is the official journal of the Voice Foundation and the International Association of Phonosurgery. Both associations are important associations dedicated to voice and laryngology research with a global reach. The Journal of Voice has a vast readership and authors from diverse backgrounds that include but is not limited to otorhinolaryngologists, laryngologists, SLPs, voice therapists, phonosurgeons, phoniatricians, voice scientists, and performing arts researchers [28]. The second most common choice was The Laryngoscope, published by Wiley. It is the official journal of the American Laryngological, Rhinological, and Otological Society, Inc. and publishes research on otolaryngology, head and neck surgery and other related medical and allied specialities. The journal has a broad global reach with readers from the USA (35%), China (8%), United Kingdom (5%), India (4%) and Australia (4%) [29]. The popularity of both journals can be attributed to their credibility and reputation for publishing evidence-based and clinically relevant research. The interdisciplinary nature of these journals provides a vast and diverse range of topics for the authors and readers. Both journals were also elected among the top journals by SLPs and otorhinolaryngologists [30].

Findings revealed a continuous increase in the number of publications, especially after the early 2000s. A closer look at the last 15 years revealed a most rapid rise in publications between 2020 and 2024. This increase is reflective of increased research interest in LPR diagnostic and treatment approaches. This rapid increase in the publication trend with a consistent annual increase of over 17 publications a year between 2021 and 2023. Although the assessment of the quality of the published research was beyond the purview of this paper, previous reviews have emphasized the need to improve the research quality of the publications. These reviews have also highlighted this increasing trend in publications on LPR [5, 20]. This increase in publications is an indirect reflection of the changing in clinical practice [9, 31, 32].

Keyword co-existence analysis highlights the interdisciplinary nature of diagnosis and management of LPR involving otorhinolaryngology, speech-language pathology and gastroenterology. The five clusters overlapped, signifying the interconnections between all the different aspects associated with LPR, such as health conditions and symptoms, voice and larynx, diagnostic techniques, management and digestive system. Also, an overlap in the usage of terminology related to GERD and LPR was noted, which could be attributed to the interchangeable use of these terms. Based on the findings of this study, future studies can be planned to provide more insight into the smaller clusters and their interconnections.

Future studies can be planned on specific topics in LPR related to diagnosis and management to track the research trends. The management of LPR includes a team-based approach, and future bibliometric analysis is needed to identify the nature and extent of involvement of multiple disciplines. Detailed thematic and content analysis of top papers in the field will identify advancements and gaps. Comparison of different geographic regions can provide region-specific trends and disparities.

The present study has some limitations, only Scopus was used for data retrieval and analysis. Studies indexed in other electronic databases and in languages other than English could have been missed out. Keyword selection and retrieval process could have led to some inconsistencies in the data and analysis. The analysis based on the counts for records does not capture the rigour, quality and relevance of the research.

## Electronic supplementary material

Below is the link to the electronic supplementary material.

Supplementary Material 1
