# Supplementary material for: Scientometric analysis of trends in research in laryngopharyngeal reflux
Source: Eur Arch Otorhinolaryngol. 2025 Apr 7;282(5):2525–32. doi: 10.1007/s00405-025-09381-1 (PMC12055652; doi:10.1007/s00405-025-09381-1)
Supplement: Supplementary file 1 — Supplementary Material 1 [file 405_2025_9381_MOESM1_ESM.docx]

**Appendix 1**

**Search string**

((ALL("reflux laryngitis") OR ALL("laryngeal reflux") OR ALL("gastropharyngeal reflux") OR ALL("pharyngoesophageal reflux") OR ALL("supraesophageal reflux") OR ALL("extraesophageal reflux") OR ALL("atypical reflux") OR ALL("laryngopharyngeal reflux") OR ALL("laryngopharyngeal reflux disease"))

**After Filters**

( ALL ( "reflux laryngitis" ) OR ALL ( "laryngeal reflux" ) OR ALL ( "gastropharyngeal reflux" ) OR ALL ( "pharyngoesophageal reflux" ) OR ALL ( "supraesophageal reflux" ) OR ALL ( "extraesophageal reflux" ) OR ALL ( "atypical reflux" ) OR ALL ( "laryngopharyngeal reflux" ) OR ALL ( "laryngopharyngeal reflux disease" ) ) AND PUBYEAR > 1956 AND PUBYEAR < 2025 AND ( LIMIT-TO ( LANGUAGE , "English" ) ) AND ( LIMIT-TO ( SRCTYPE , "j" ) OR LIMIT-TO ( SRCTYPE , "p" ) ) AND ( LIMIT-TO ( DOCTYPE , "ar" ) OR LIMIT-TO ( DOCTYPE , "re" ) OR LIMIT-TO ( DOCTYPE , "cp" ) )
